# Relapse Risk After Mandated Inpatient Substance Use Rehabilitation: Implications for Post-Discharge Policy and Monitoring

**DOI:** 10.1192/bjo.2026.11200

**Published:** 2026-06-30

**Authors:** Faycal Ikhlef, Nirvana Chandrappa, Majid Alabdulla, Wesam Smidi, Ahmad Alater

**Affiliations:** Hamad Medical Corporation, Doha, Qatar

## Abstract

**Aims:**

Relapse following discharge from inpatient rehabilitation remains a major challenge in substance use disorder (SUD) care and is associated with significant clinical, social, and legal consequences. While relapse rates are well described, far less is known about the timing of relapse, particularly within mandated treatment systems where patients are referred through legal (Court) or regulatory pathways (General Persecution) and experience structured supervision and monitoring. Identifying periods of heightened vulnerability following discharge is critical for optimising aftercare strategies and allocating resources effectively. This study aimed to characterise the timing and distribution of relapse events following discharge from inpatient SUD rehabilitation in Qatar and to identify critical post-discharge periods for targeted preventive intervention within mandated treatment pathways. We hypothesised that relapse risk would be highest during the early post-discharge period.

**Methods:**

A retrospective cohort study was conducted including 72 patients discharged from an inpatient SUD rehabilitation programmeoperating under a mandated treatment framework. All patients entered a structured aftercare pathway with routine follow-up and urine drug screening. Relapse was defined as a documented positive urine drug screen for illicit substances during follow-up. Time-to-relapse was measured from the date of discharge and tracked over a 26-week post-discharge period. Patients who did not relapse during follow-up were censored at their last negative drug screen. Kaplan–Meier survival analysis was used to estimate relapse-free survival and to describe the temporal distribution of relapse events across the follow-up period.

**Results:**

During the 26-week follow-up, 40.3% of patients (n=29) experienced relapse. The median time-to-relapse was 28 days, indicating that half of all relapse events occurred within the first four weeks following discharge. Relapse timing ranged from 7 to 182 days, demonstrating substantial inter-individual variability in post-discharge recovery stability. Kaplan–Meier survival curves showed the steepest decline in relapse-free survival during the first month after discharge, identifying this period as the highest-risk phase for recurrence. Beyond the initial four weeks, the hazard of relapse declined progressively over time, with fewer new relapse events observed in later follow-up.

**Conclusion:**

This time-to-event analysis demonstrates that the first four weeks following discharge constitute a critical vulnerability window for relapse among patients undergoing mandated inpatient SUD rehabilitation. Findings support prioritising intensive early aftercare, enhanced monitoring, and rapid-response interventions during the immediate post-discharge phase. Targeting this high-risk period within mandated treatment pathways may reduce early relapse and improve longer term recovery outcomes.

